# A forgotten parasitic infestation in an immunocompromised patient-a case report of crusted scabies

**DOI:** 10.11604/pamj.2020.36.238.24288

**Published:** 2020-08-04

**Authors:** Martin Agyei, Afua Ofori, Elliot Koranteng Tannor, John Jude Annan, Betty Roberta Norman

**Affiliations:** 1 Dermatology Unit, Department of Medicine, School of Medicine and Dentistry, Kwame Nkrumah University of Science and Technology, Kumasi, Ghana,; 2 Department of Medicine, Komfo Anokye Teaching Hospital, Kumasi, Ghana,; 3 Department of Obstetrics and Gynaecology, School of Medicine and Dentistry, Kwame Nkrumah University of Science and Technology, Kumasi, Ghana

**Keywords:** Crusted scabies, *Sarcoptes scabiei var hominis* mite, neglected tropical disease, immunocompromised

## Abstract

Crusted scabies is a rare and highly contagious form of Sarcoptes scabiei var hominis infestation whose incidence may increase in the near future due to increasing use of immunosuppressive therapies and a general lack of awareness about the condition. It is misdiagnosed as psoriasis, irritant dermatitis or eczema. Delays in diagnosis lead to widespread transmission amongst contacts leading to potential community outbreak. Crusted scabies is extremely difficult to treat and there are growing concerns of possible resistance to current treatment. This case report describes a 44-year-old Ghanaian woman with human immunodeficiency virus (HIV) infection and diagnosed with skin scrapings. Treatment was initiated but the patient died from HIV related complications. Crusted scabies, though rare, should be an issue of global concern due to the potential for widespread dissemination. Adequate resources need to be channeled into scabies eradication as well as education of health personnel to promptly identify and treat cases.

## Introduction

Scabies is a highly contagious and intensely pruritic skin infestation caused by the *Sarcoptes scabiei var hominis mite*. It is also known as the ‘seven-year itch’ due to its persistent nature of infestation without treatment. Scabies has been in existence for centuries with documented cases dating as far back as Aristotle (384 BC to 322 BC). Crusted (Norwegian) scabies is found in people with compromised immune system as well as persons with decreased sensory functions as in leprosy. The first case of crusted scabies was reported amongst Norwegian lepers in 1848 hence the name Norwegian scabies [1]. With the emergence of human immunodeficiency virus (HIV) in 1981 and the introduction of various immunosuppressive therapies, crusted scabies is becoming an issue of global concern. Unfortunately, most clinicians misdiagnose cases as irritant dermatitis, psoriasis or eczema and prescribe topical steroids which further compounds the problem. In instances where patients are accurately diagnosed, treatment also poses a challenge due to increasing drug resistance among other factors [2]. This case report serves as a reminder to physicians to consider crusted scabies in ‘difficult to treat’ hyperkeratotic skin lesions especially in immunocompromised individuals. Such patients should be referred to dermatologists without delay. It also provides the current treatment options available for this condition.

## Patient and observation

**Figure 1:**
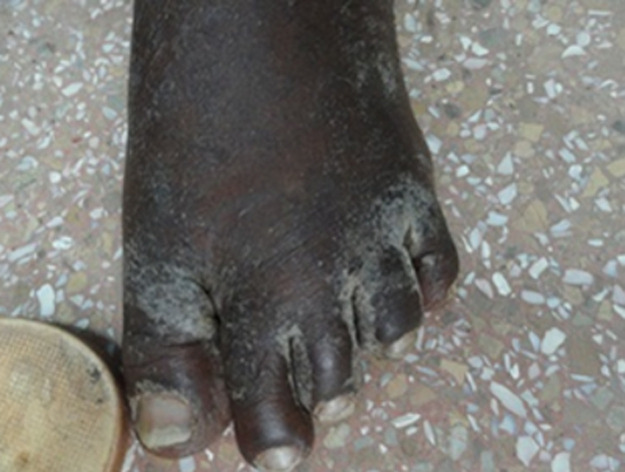
showing the crusted scabies in the web of the feet

**Figure 2:**
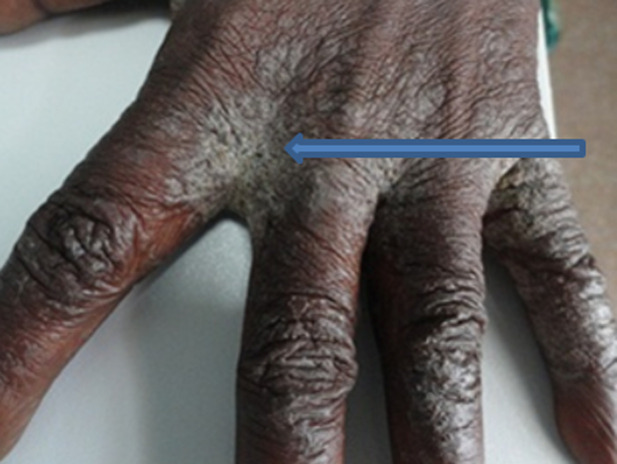
showing scabies in web of the hand with also thick scales

**Figure 3:**
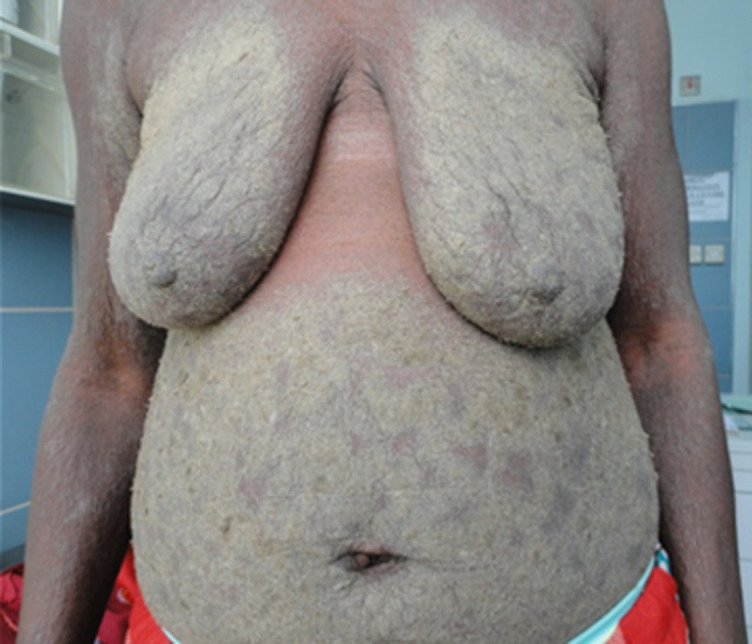
showing scabies on the abdomen and breasts

**Figure 4:**
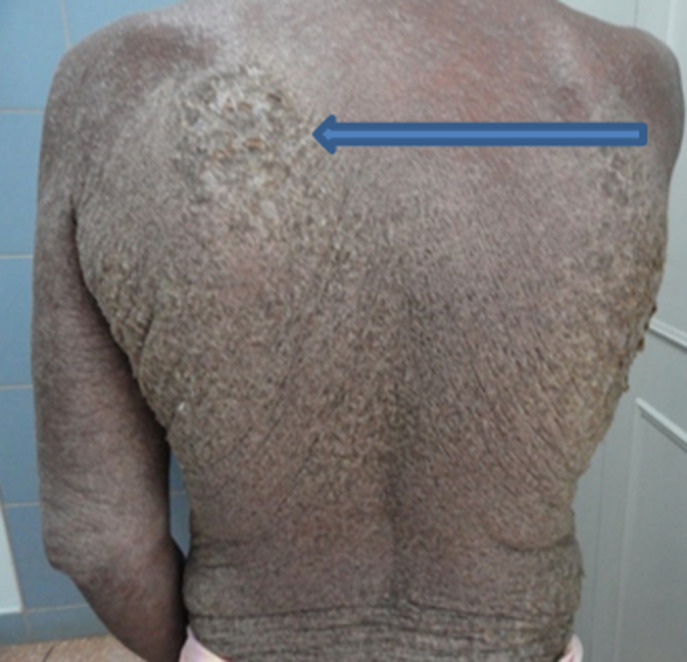
showing thick scales on the back (blue arrow)

A 44-year-old Ghanaian lady had an extensive excruciatingly itchy papulosquamous rash for two years. She presented to several general physicians who treated her for a non-specific irritant contact dermatitis using topical steroids and other proprietary measures but to no avail. Her failure to respond to the various therapies prompted a doctor to test her for human immunodeficiency virus (HIV) infection six months prior to her referral and tested positive to HIV-1 antibodies. Anti-retroviral therapy had not been initiated yet when she was referred to our tertiary hospital to see a dermatologist. The lesions had started as papules and vesicles in the webs of her fingers and later spread to involve her trunk and the rest of her body. Her daughter as well as her mother had similar pruritic vesicular eruption but less extensive. On physical examination, virtually the entire skin was covered with thick crusted scales (Figure 1, Figure 2, Figure 3, Figure 4). She was diagnosed with crusted scabies and was sent to the laboratory for skin scraping and microscopy which showed high parasitaemia of *Sarcoptes scabiei* mites and eggs. She was given a stat dose of 200mcg/kg body weight of Ivermectin orally and 10mg cetirizine daily as an outpatient. She applied petroleum jelly as an emollient. She was to repeat the Ivermectin at weekly intervals whilst monitoring her response. Amoxicillin/clavulanic acid 625mg twice daily for seven days and potassium permanganate baths were also prescribed for superimposed bacterial infection. She was apparently responding to therapy with improved skin lesions when she had explosive diarrhoea at home. The family tried to treat her at home and did not seek medical assistance. She died at home.

## Discussion

Scabies is a parasitic infestation by the female *Sarcoptes scabiei var hominis* mite. Distribution is worldwide with an estimated global incidence of 300 million with Indonesia, China, Timor-Leste, Vanuatu and Fiji having the greatest burden of disease. Individuals of all age groups can be affected but it tends to occur more frequently among the elderly, adolescents and young children [3]. Spread is aided by close contact and predisposing factors include overcrowding, immune-compromised status, malnutrition, poor hygiene, multiple sexual partners and late diagnosis [4]. Infestation with the 0.3 to 0.5mm long female mite is initiated by close skin contact of about 10 to 15 minutes during which the mite is transferred to the new host. The mite tunnels into the stratum corneum of the skin depositing faeces and about 3 eggs per day. These eggs are transformed into larvae within 3 to 10 days and then subsequently into nymphs that mature into adults. Following mating, the male dies but the fertilized female lives for about 4 weeks depositing eggs.

Symptoms of infestation, manifested by itching, are mediated by an allergic reaction to the mite´s faeces deposited in the burrows. Previously exposed individuals tend to develop symptoms within days due to prior sensitization. First time infestations may take 4 to 6 weeks as they mount an immune-mediated antigen-specific delayed hypersensitivity reaction [5]. Classical scabies (also known as simple scabies) affects the web spaces in-between the digits, volar aspect of wrist, elbows, breasts and external genitalia with the scalp, face, neck, palms and soles usually spared; these areas may be involved especially in young children and immunocompromised. Persistent itching is characteristic and is often worse at night and following a hot bath. Diagnosis in most resource poor settings is usually clinical and is based on history, the classical itch, morphology and distribution of lesions. Itch among close contacts heightens the suspicion. Microscopic examination of a skin snip mounted in potassium chloride may reveal the mites or their eggs and is the definitive diagnosis. The ink test, where the mite burrows are outlined with ink can also be performed. Although cheap, in both instances a negative test does not rule out infestation since the classical infestation involves only about 10 to 15 mites. Polymerase chain reaction (PCR) can be used to confirm the presence of mites from scales and so can epiluminescence microscopy but are not widely available especially in developing countries [4].

Crusted scabies is an extremely contagious and fortunately, a rarely encountered form of scabies. In crusted scabies, the skin is infested with thousands to millions of mites, unlike in classical scabies where infestation is limited to only a few number, with the formation of thick scaly or hyperkeratotic crusts hence the name crusted scabies. The highly contagious lesions may or may not be pruritic and no burrows are visible. Unlike classical scabies, it can involve all skin areas including the scalp, palms and soles whilst the nails show hyperkeratosis and thickening. Commonly, there is relative sparing of the face but there are cases where the face is also extensively involved which was the case in our patient. The immunocompromised, malnourished, glucocorticoid users and patients with neurotic or psychiatric illnesses that limit scratching tend to present with this form of scabies since they are unable to mount an immune response or scratch respectively [6]. Some cases have however been reported in immunocompetent people. In our patient, HIV was the predisposing factor. There are case reports amongst individuals with lepromatous leprosy due to defective T-cell mediated immunity [7].

The immune response in classical scabies is dominated by a T helper 1-type cytokine profile associated with CD4 T-lymphocytes, whereas a non-protective T helper 2-type cytokine profile is present in crusted scabies with CD8 lymphocytes as the predominant effector cells. Interferon-γ/interleukin (IL)-4 ratio is higher in S. scabiei-stimulated peripheral blood mononuclear cells (PBMCs) from classical scabies patients than in PBMCs from crusted scabies patients. Increased levels of IL-5 and IL-13 are observed in stimulated PBMCs from crusted scabies as compared with PBMCs from classical scabies patients. Eosinophil counts are highly elevated in crusted scabies whereas moderate elevations are seen in the classical form. The tissue damage in crusted scabies is caused by mostly CD8 mediated direct cytotoxicity against keratinocytes and the release of cytokines, which amplify the inflammatory response by targeting resident skin cells [8]. Crusted scabies must be distinguished from conditions such as disseminated fungal infection, allergic contact dermatitis, psoriasis and eczema which may present similarly [6]. Diagnosis was confirmed by the presence of increased numbers of the mites and their eggs on light microscopy. Delays in the diagnosis of crusted scabies has dire consequences as healthcare personnel can get infested and transmit the mites to other patients who will in turn infest their family and other close contacts.

Complications of scabies such as secondary bacterial infection of burrows by *Staphylococcus aureus* and *Streptococcus pyogenes* may occur especially in the tropics. Rheumatic fever and post streptococcal glomerulonephritis with renal failure may complicate *S. pyogenes* superinfection particularly in children [8]. Our patient died in the course of treatment but this was most likely a result of HIV related complications rather than a treatment complication. Sepsis from superimposed bacterial infection though considered, is a remote possibility in our patient since she was on Amoxicillin/clavulanic acid for primary prevention of bacterial infection. Treatment of Norwegian scabies poses a big challenge due to the large number of mites, hyperkeratotic skin that is difficult to penetrate with topical scabicides, and the large number of infected contacts due to its highly contagious nature. Several therapies have been tried including repeated application of scabicides, use of oral ivermectin alone or a combination of the two therapies.

Topical agents are generally considered to constitute first-line treatment of classical scabies, with oral ivermectin being reserved for recurrent, difficult-to-treat cases, or crusted scabies. Most authorities however recommend the use of both topical and systemic agents simultaneously [9]. Various scabicides such as 5% permethrin cream, 1% lindane cream, 5 to10% sulphur and malathion are available. Some authorities recommend the use of keratolytic agents such as 6% salicylic acid prior to application of scabicides to allow better penetration. Galenicals and home based treatments such as neem and tea tree oil have been used with varying success. Scabicides should be applied to all skin areas after bathing and left in contact with the skin for 24 hours. Finger and toe nails should also be trimmed and the topical scabicide applied to the subungual area as this site tends to harbour mites for re-infestation.

All close contacts need to be treated simultaneously even if asymptomatic and all clothes and personal effects washed with hot water. Alternatively, these can be kept from human contact for at least three days since the mite is unable to survive outside the human host beyond this period, except in the case of crusted scabies where the mites can survive for up to a week in the environment, feeding on the crusted stratum corneum. Antihistamines, salicylates, and calamine lotion may be used to relieve itching during treatment. Failure of resolution of symptoms may signify treatment failure or re-infestation and the need for retreatment with careful tracking of all close contacts to ensure they are adequately treated. There is concern about the development of resistance to the currently available agents [9, 10]. Topical agents currently available have the tendency to cause ‘post-scabietic’ eczema and cannot be used when the skin is damaged due to irritation and a potential for toxicity due to systemic absorption. In such instances ivermectin is the only option but ironically cannot be used in children below the age of five years. There have also been reports of ivermectin resistance [9]. There is the need to research into new agents since crusted scabies can pose a significant public health threat.

## Conclusion

Crusted scabies, though rare, should be an issue of global concern due to the potential for widespread dissemination. Adequate resources need to be channeled into scabies eradication and training of health personnel to promptly identify and treat patients.
